# Basic Resources and Psychological Strengths as Predictors of Depressive Symptoms

**DOI:** 10.1093/geroni/igab046.418

**Published:** 2021-12-17

**Authors:** Charikleia Lampraki, Daniela Jopp

**Affiliations:** University of Lausanne, Lausanne, Vaud, Switzerland

## Abstract

Loss of personal resources is expected to have a negative effect on well-being in all ages, however, in very old age, this effect may be exacerbated. Centenarians, who are confronted with accumulated age-related losses, may be at higher risk of experiencing depressive symptoms. This study investigated the link between basic resources (i.e., health, social network) and depressive symptoms and whether it was mediated by psychological strengths (i.e., meaning, optimism) in 119 centenarians and near-centenarians (Mage = 99.7 years). Results indicated that meaning in life fully mediated the link between health and depressive symptoms, and the link between social network size and depressive symptoms. Similarly, optimism mediated the link between network and depression, but no mediation effect was found when considering health as basic resource. In sum, basic resources are only indirectly associated to depressive feelings, with psychological strengths playing an important intervening role in very old age.

